# Prize Essays

**Published:** 1845-02

**Authors:** 


PRIZE ESSAYS.

The committee appointed to award the premium for the best essay on the *Health of the Clergy* not being able to agree as to which was the best, decided to divide the prize between the authors of the two having the highest claims. It was accordingly awarded to Dr. Sutton, of Kentucky, and Dr. Cogley, of Indiana, in equal sums. The successful essays have been published in the Protestant & Herald, and will probably be issued in pamphlet form. Y.

